# The Influence of Moisture Content and Temperature on the Long-Term Storage Stability of Freeze-Dried High Concentration Immunoglobulin G (IgG)

**DOI:** 10.3390/pharmaceutics12040303

**Published:** 2020-03-27

**Authors:** Arnold Duralliu, Paul Matejtschuk, Paul Stickings, Laura Hassall, Robert Tierney, Daryl R. Williams

**Affiliations:** 1 Surfaces and Particle Engineering Laboratory, Department of Chemical Engineering, Imperial College London, SW7 2AZ, UK; a.duralliu15@imperial.ac.uk; 2 Standardisation Science, National Institute for Biological Standards and Control, Blanche Lane, South Mimms, Potters Bar, Hertfordshire, EN6 3QG, UK; Paul.Matejtschuk@nibsc.org; 3 Bacteriology Division, National Institute for Biological Standards and Control, Blanche Lane, South Mimms, Potters Bar, Hertfordshire, EN6 3QG, UK; Paul.Stickings@nibsc.org (P.S.); Laura.Hassall@nibsc.org (L.H.); Robert.Tierney@nibsc.org (R.T.)

**Keywords:** freeze-drying, Immunoglobulin G, concentration, stability, monomer, binding activity, moisture, reconstitution

## Abstract

High protein concentration products for targeted therapeutic use are often freeze-dried to enhance stability. The long-term storage stability of freeze-dried (FD) plasma-derived Immunoglobulin G (IgG) from moderate to high concentrations (10–200 mg/mL) was assessed. Monomer content, binding activity and reconstitution times were evaluated over a 12-month period under accelerated and real-term storage conditions. In the first case study it was shown that FD IgG from 10 to 200 mg/mL had minimal monomer/activity losses at up to ambient temperature after 12 months of storage. However, at 45 °C the sucrose-to-protein ratio played a significant impact on IgG stability above 50 mg/mL. All IgG concentrations witnessed moisture ingress over a 12-month period. The impact of moisture ingress from environmental exposure (between 0.1% and 5% *w*/*w* moisture) for IgG 50 mg/mL was assessed, being generated by exposing low moisture batches to an atmospheric environment for fixed time periods. Results showed that at −20 °C and 20 °C there was no significant difference in terms of monomer or antigen-binding activity losses over 6 months. However, at 45 °C, there were losses in monomer content, seemingly worse for higher moisture content samples although model binding activity indicated no losses. Finally, the difference between a low moisture product (0.1–1% *w*/*w*) and a moderately high moisture (3% *w*/*w*) product generated by alternative freeze-drying cycles, both stoppered under low oxygen headspace conditions, was evaluated. Results showed that at −20 °C and 20 °C there was no difference in terms of binding activity or monomer content. However, at 45 °C, the low moisture samples had greater monomer and binding activity losses than samples from the highest moisture cycle batch, indicating that over-drying can be an issue.

## 1. Introduction

There is rising demand in the biopharmaceutical industry for increasingly high concentrations of therapeutic proteins, such as monoclonal antibodies (mAbs). However, with this requirement there comes a host of challenges, including an increased instability, degradation and viscosity, as well as unwanted protein–protein interactions in the liquid state [1,2]. There is no precise standardized definition of what constitutes a “high concentration” protein formulation but generally it has been considered to fall between 50 and 150 mg/mL [3]. Freeze-drying (FD) is a common technique used to enhance shelf stability by delivering a product in a solid-state format. [4,5]. It consists of three stages: (1) Freezing (solidification), (2) primary drying (sublimation of frozen ice) and (3) secondary drying (desorption of bound water). Sugar-based excipients are usually added to these formulations to enhance the stability of the active pharmaceutical ingredient (API) during prolonged storage. Other studies have focused their investigations into the slow reconstitution times of high concentration protein formulations and ways to improve them [6,7]. However, in addition to the continuing development of higher protein concentration formulations in FD formats, there are challenges with regard to selecting the appropriate moisture content for long-term storage stability. Typically, after FD the resulting solid freeze-dried “cake” would be expected to have a low moisture content. For FD intravenous Immunoglobulin G formulations, the acceptable moisture contents would be at or below 3% *w*/*w* [8]. Plasma-derived immunoglobulin has long been used as a therapeutic product and intravenous immunoglobulin concentrations of 50–100 mg/mL are common [9,10]. Previous studies have also looked into the stability of mAbs with regard to higher moisture content ranges of between 1% and 8% *w*/*w*; however, these studies focussed on the impact of the higher end of the moisture range. Breen et al. [11] was able to show that while increasing the moisture content up to 8% *w*/*w* might decrease chemical stability in FD rhuMAb, it might in fact actually increase the physical stability as long as storage was below the dry state glass transition (T_g_). The range of moisture content that is desirable in FD cakes has long been a matter of debate. Hsu et al. showed that an optimum moisture content range might exist, whereby both over and under drying could be detrimental to stability [12]. Long ago Greiff hypothesised that the relationship between moisture content and stability could correspond to a “bell shaped” distribution [13]. Alternatively, Pikal et al. noted that for FD Human Growth Hormone at 40 °C, there was a linear relationship between moisture content and aggregation rather than a bell-shaped distribution [14]. Guidelines for use when developing stressing strategies for biotechnology products have been published [15] and industrial stressing conditions have also been reviewed [16,17]. During long-term storage, the sugar excipient used in the formulation, moisture content and temperature can all affect the physical and chemical stability of FD cakes. Plasma-derived IgG was chosen to be investigated here as a substitute model for a typical therapeutic mAbs because of its relative ease of availability at high concentrations. A comprehensive set of long-term case studies investigating the stability of FD IgG was assessed over a 6–12-month period. The monomer content, retention of anti-diphtheria/tetanus antibody titres and change in moisture content were evaluated under accelerated and real-time storage conditions (−20 °C, ~ <5% RH; 20 °C, ~40% RH; and 45 °C, ~10% RH, though the incubators used did not directly control the RH) to assess FD storage stability.

## 2. Materials and Methods

### 2.1. Materials and Formulation Characterisation

The bulk of the 50 mg/mL IgG (2.2 L) was obtained by dialysis from time-expired clinical grade standard product (NIBSC, Potters Bar, UK) and maintained sterile at 2–8 °C. A quantity, 150 mL, was dialysed into 1% *w*/*v* sucrose, 10 mM citric acid adjusted at pH 6.6 with 5 M NaOH and 0.01% Tween 20 against 3 × 5 L over 20 h using a Spectrapor 8 kDa cut-off dialysis membrane (Sigma-Aldrich, Gillingham, UK). A portion from this bulk was diluted down to 10 mg/mL. The remaining bulk pool was then ultrafiltered to get nominal target concentrations of 100 and 200 mg/mL IgG. All the preparations were then dialysed in dialysis membrane sacs against the citrate Tween 20 sucrose buffer. Target concentrations were achieved by diafiltration using the Vivaflow 200 crossflow system and with a 50 kDa PES (polyethyl sulfone) membrane cartridge (Sartorius Stedim Biotech, Gottingen, Germany). Concentrations were confirmed by OD 280 nm (E1 % for IgG = 13.5 AU) using triplicate 1:100 dilutions measured with a UV spectrophotometer (Pharmacia Biotech, GE Healthcare, Little Chalfont, UK) blanked on sucrose citrate buffers. Average readings were 0.98 ± 0.02%, 5.43% ± 0.19%, 9.86% ± 0.13% and 20.4% ± 0.97% *w*/*v*. These were approximated to 1%, 5%, 10% and 20% w/v, which is equivalent to 10, 50, 100 and 200 mg/mL, respectively.

### 2.2. Modulated DSC

Solid sample or liquid aliquots of IgG were loaded into large volume hermetically sealed pans (part number 900825.902 TA Instruments, Elstree, UK). Samples were evaluated on Q2000 DSC (TA Instruments) against an empty pan as reference. A heating ramp rate at 5 °C/min up to 200 °C was used. Instrument calibration was performed using an indium sample of known mass. Data analysis was performed using Universal Analysis Software (TA Instruments).

### 2.3. Case Study 1: Freeze Drying of a High Concentration IgG

A batch using a “low” moisture cycle run was included in all the IgG concentrations (10–200 mg/mL). Sample filling was completed with an automated multipette stream (Eppendorf, Stevenage, UK) into 5 mL volume screw capped vials (41.5 × 18 mm i.d. Schott VC005, Adelphi Tubes, Haywards Heath, UK). The fill volume for all concentrations was 1 mL. The vials were loaded onto the shelves of the Telstar LyoBeta 15 (Azbil-Telstar SpA, Terrassa, Spain). After the cycles had finished the vials were backfilled with dry N_2_ and were stoppered down with 14 mm diameter igloo halobutyl stoppers (Adelphi Group, Haywards Heath, UK). Afterwards the vials were screw-capped, labelled and stored at −20 °C, 20 °C and 45 °C until further testing.

### 2.4. Case Study 2: Optimum Moisture Content of IgG

A total of 50 mg/mL IgG was lyophilised with the same conditions and same “low” FD cycle as described in Section 3.2. After FD, samples were laboratory-stored at 20 °C (with a relative humidity of 57% ± 2% RH, as measured with a TFH 620 Hygrothermometer (Ebro-Xylem, Ingoldstat, Germany). The stoppers were removed temporarily, and vials were exposed to the moisture in the external atmosphere for different time intervals (0, 30, 90 and 180 min) to result in different moisture contents in the cakes. A total of 4 different moisture contents were prepared and an average confirmed using Karl Fisher titration in triplicate (*n* = 3).

### 2.5. Case Study 3: Comparison of Low and High Moisture FD Cycles

The “high” moisture cycle and “low” moisture cycle batches of FD IgG (50 mg/mL) were prepared to allow comparison between these two different cycles that induce moisture content as shown in Table 1. After FD cycles, samples were once again backfilled from vacuum with dry N_2_ and were stoppered down with 14 mm diameter igloo halobutyl stoppers (Adelphi Group, Haywards Heath, UK). Afterwards the vials were screw-capped, labelled and stored at −20 °C, 20 °C and 45 °C until further testing.

### 2.6. Diphtheria and Tetanus ELISA

Anti-diphtheria or anti-tetanus IgG binding activity levels are commonly determined using the ELISA technique as an alternative to in vivo toxin neutralization tests [18]. In brief, 96-well plates were coated with either diphtheria toxoid (NIBSC product code 13/212) or tetanus toxoid (NIBSC product code 02/126) as coating antigen, sealed and left to incubate overnight at 4 °C. The next day plates were washed with PBS containing Tween-20 0.05% *v*/*v* (PBST) and then blocked with blocking buffer (PBST + 5% skimmed milk powder). Plates were incubated at 37 °C for 1 h and washed as before. Sample and reference antitoxin dilutions were prepared in sample buffer (PBST + 1% skimmed milk powder). WHO International Standard antitoxins for diphtheria (NIBSC product code 10/262) and tetanus (NIBSC product code TE-3) were included on each plate to allow specific IgG concentrations to be expressed in IU/mL. A total of 200 mL of diluted sample or reference were added to the top row of wells and titrated down the plate using a manual multichannel pipette set to 100 µL. After serial dilutions, plates were once again incubated at 37 °C for 2 h and washed as before. Anti-human IgG HRP-conjugate (Sigma A8792, Sigma-Aldrich) was diluted 1/2000 in sample buffer and 100 µL was added to each well before incubation at 37 °C for 1 h. Substrate solution containing citric acid buffer and ABTS (2,2′-Azinobis (3-ethylbenzothiazoline-6-sulfonic acid) diammonium salt) tablets was added to all wells for colour to develop. Absorbance was read at 405 nm on a Molecular Devices Vmax plate reader running Softmax Pro 6.5.1 (Molecular Devices, Wokingham, UK). Data analysis was performed in CombiStats (version 5.0, 2013, EDQM, Strasbourg, France).

### 2.7. SEC-HPLC

Size-exclusion high performance liquid chromatography (SEC-HPLC) is a commonly applied chromatographic method used for determining of aggregation or monomer content of biotherapeutic proteins in solutions via size or molecular weight differences [19]. SEC-HPLC was used to determine the monomer content of the FD IgG samples after reconstitution. Analysis was performed using the Thermo Scientific UltiMate 3000 HPLC System (Thermo Fisher Scientific, Loughborough, UK) with a TSKgel G3000SWXL HPLC column (300 × 7.8 mm, Sigma-Aldrich). Ultraviolet (UV) detection was measured at 280 nm in triplicate (*n* = 3). The mobile phase was prepared from the formula as outlined in European Pharmacopeia 9.0: Human Normal Immunoglobulin for intravenous administration (01/2012:0918) [8], consisting of disodium hydrogen phosphate dihydrate (0.49% *w*/*v*), sodium dihydrogen phosphate monohydrate (0.17% *w*/*v*), sodium chloride (1.17% *w*/*v*) and sodium azide (0.01% *w*/*v*). The flow-rate on the apparatus was set at 0.5 mL/min. Peak analysis was performed using Chromeleon 7.2SR software (Thermo Fisher Scientific, Loughborough, UK).

### 2.8. Residual Moisture Content

The moisture content of the FD cakes within the vials was measured using an automated coulometric Karl Fischer instrument (Mitsubishi CA-200, A1-Envirosciences Ltd., Blyth, UK). Samples were transferred into HPLC autosampler vials within a pyramid dry bag (Captair pyramid, 2200A Cole Palmer, London, UK). Nitrogen gas was used to purge the pyramid air bag to ensure that a humidity < 5% RH was achieved. All samples were tested in triplicates (*n* = 3), i.e., 3 vials were tested.

## 3. Results and Discussion

### 3.1. Case Study 1: Effect of Moisture Ingress and Excipient Ratio on Storage Stability of High Concentration IgG

**Figure 1:**
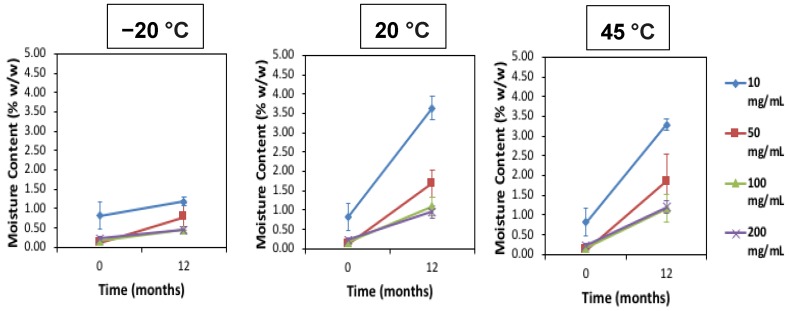
Average moisture content (%) for low to high concentration freeze-dried (FD) IgG before and after 12 months of storage at −20, 20 and 45 °C. Error bars are 95% confidence intervals (*n* = 3 vial replicates).

Polyclonal human immunoglobulin is derived from many thousands of donors from the population, and so a dimer content of between 8% and 10% can occur naturally and is of itself not an indicator of aggregation [20]. Usually a protein-to-sugar weight ratio of 1:1 would be suitable for optimal stabilisation. However, in this study the IgG molar/weight ratio was intentionally varied as the sucrose excipient concentration of 10 mg/mL (1% *w*/*w*) was maintained in order to investigate the destabilizing effect of moisture ingress and/or storage temperature conditions during long-term storage. As such the sucrose:protein molar ratio for these investigations were as follows: 1% sucrose to 1% IgG is 439:1; 5% IgG is 88:1; 10% IgG is 44:1; and 20% IgG is 22:1. The moisture content before and after 12 months storage is displayed in Figure 1. 

After 12 months of storage, for all IgG concentrations there was an increase in moisture content, especially at the higher storage temperatures of 20 °C and 45 °C. The 10 mg/mL samples had the highest moisture ingress at 20 °C and 45 °C at around 3–4% *w*/*w* moisture content (*n* = 3). With increasing IgG concentration there was less moisture uptake at these elevated temperatures of between 1% and 2% *w*/*w* moisture. The most common sources of moisture ingress are likely due to either trapped moisture in the headspace of the vials or moisture actually emanating from the rubber stoppers [14,21]. Past studies and research have shown the negative consequences of elevated moisture and temperature on FD biologics stability and structure [22,23]. Increasing moisture content can lead to a reduction of the T_g_ due to the its plasticizing effect [24,25]. Table 2 shows the T_g_ of the IgG after 12 months of storage as measured with DSC (*n* = 2). The difference between storage temperature and the T_g_ (denoted as T_g_-T) is largest at the lowest temperature of −20 °C storage and becomes smaller as the storage temperature is increased through 20 °C to 45 °C.

**Figure 2:**
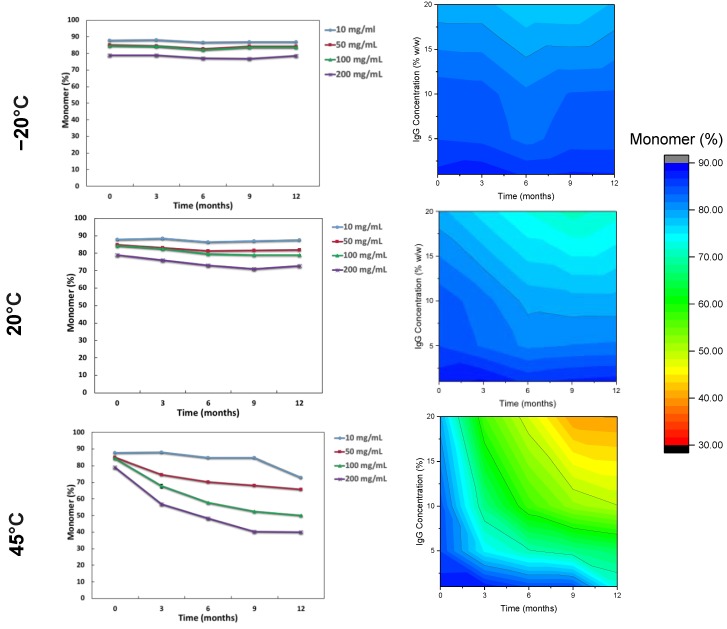
Average monomer content (%) over time for low to high concentration IgG with contour stability maps as measured by SEC-HPLC over 12 months stored at (from top to bottom) −20 °C, 20 °C and 45 °C (*n* = 3 vial replicates). Error bars at 95% confidence intervals are too small to be seen.

**Figure 3:**
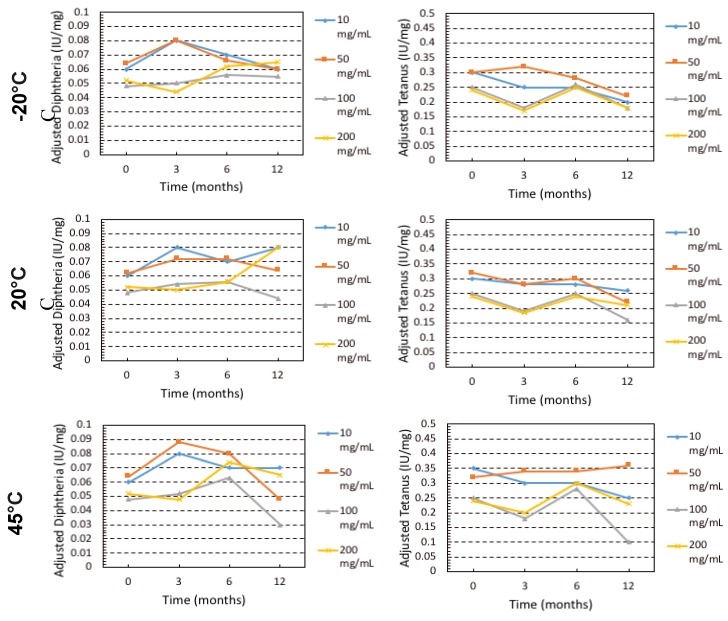
ELISA Diphtheria and Tetanus normalized binding activity (IU/mg) for IgG over the course of 12 months stored at −20 °C, 20 °C and 45 °C.

The impact of the storage conditions and moisture on the monomeric (%) content and binding activity of the IgG are shown in Figure 2 and Figure 3. Storage at −20 °C showed that there was minimal monomeric loss (less than 1%) for IgG at all concentrations. Increasing temperature resulted in greater loss of monomer and higher IgG concentrations suffered greater losses in monomer, especially at 45 °C. The order from least monomer loss to highest at 45 °C storage was 10 mg/mL < 50 mg/mL< 100 mg/mL < 200 mg/mL. The binding activity (IU/mL normalised into IU/mg) was measured by ELISA for specific IgG (anti-diphtheria andante- tetanus) over the course of the 12-month storage. Anti-tetanus and anti-diphtheria antibodies were chosen as markers for specific IgG since the majority of donors used in production of the IgG products will have received diphtheria and tetanus immunisation as part of routine immunisation programmes. It is interesting to note that while the monomer content was decreasing throughout storage at elevated temperature, the binding activity remained relatively stable until the last time point (T = 12) and only for the 50 and 100 mg/mL samples. It is important to note that the specific antibodies being measured here represent only a small proportion of the total IgG in the formulation. However, for the 200 mg/mL samples there was high variability in activity, due to fact that the 200 mg/mL samples did not fully reconstitute, thus giving variable ELISA results. 

**Figure 4:**
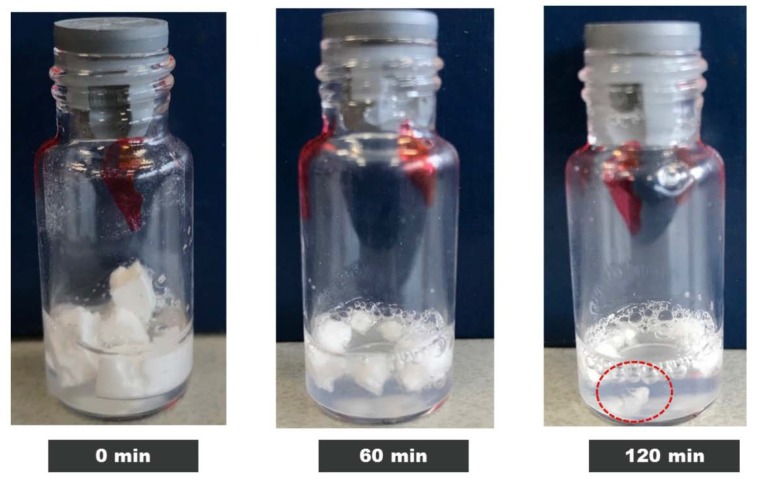
Visual appearance of IgG 200 mg/mL dissolved in water up to 0, 60 and 120 min for the T = 12-month-old sample aged at 45 °C.

The 10, 50 and 100 mg/mL IgG had a clear visual appearance after being fully reconstituted. However, the 200 mg/mL samples stored at 45 °C were slightly cloudy/viscous with tiny clumps of particulates still undissolved (Figure 4). In addition, on average the 10–50 mg/mL samples took up to 5 min to reconstitute while the 200 mg/mL samples took over 2 h to dissolve. Long reconstitution times have been commonly reported for high concentration protein solutions [26,27], and strategies for addressing this have been shared [7].

The results presented in this study are slightly unusual in that the highest concentration protein was shown to be the least stable. These results are counter to the general trend expecting higher concentrations tending to be more stable. It is possible that at these very high concentrations of FD IgG they are more temperature labile compared to lower concentrations due to a lack of excipient protection from the formulation, as the ratio of IgG:sucrose falls from 400:1 to 22:1. Table 2 shows that the 10 mg/mL IgG had a smaller difference (T_g_-T) value than for the higher concentrations, yet demonstrated lower monomer loss at 45 °C. Even though the lowest concentration IgG had the highest moisture content and reduced T_g_, it was shielded because it had a higher excipient-to-protein ratio protection while the higher concentration did not, thus supporting a case for water replacement theory. However, the moisture content was similar at both 20 °C and 45 °C, yet the largest drops were only observed at 45 °C for samples of 50–200 mg/mL. One possible explanation for this is that of the T_g_ being closer to the high storage temperature and hence increasing molecular flexibility—supporting a role for the glass dynamics hypothesis (T_g_-T). The question of optimum moisture content and mechanisms of stabilization (be it water substitution theory or glass vitrification hypothesis) has been a matter of debate [28]. These results provide a case for both water replacement theory and vitrification hypothesis in terms of stabilization. Overall, moisture ingress during storage (from as high as 1% to 4% *w*/*w*) occurs for all FD IgG concentrations up to 200 mg/mL and needs to be considered, although for these high concentration proteins the moisture appeared to have minimal impact on the stability at −20 °C and 20 °C.

### 3.2. Case Study 2: Optimum Moisture Content of IgG

**Figure 5:**
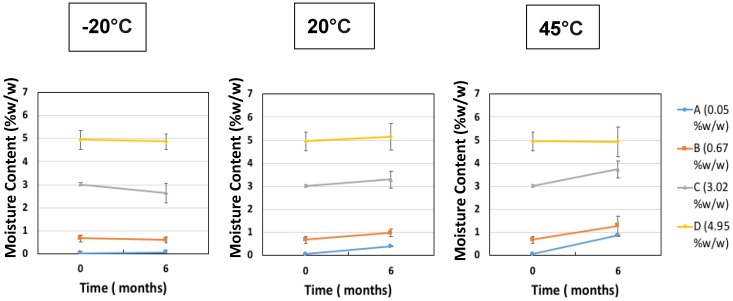
Change in moisture content (% *w*/*w*) after 6 months of storage starting at different initial starting moisture samples of IgG 50 mg/mL (A = 0.05% ± 0.01% *w*/*w*, B = 0.67% ± 0.14% *w*/*w*, C = 3.02% ± 0.06% *w*/*w* and D = 4.95% ± 0.40% *w*/*w*) at −20 °C, 20 °C and 45 °C. Error bars represent 95% confidence intervals (*n* = 3 vial replicates).

**Figure 6:**
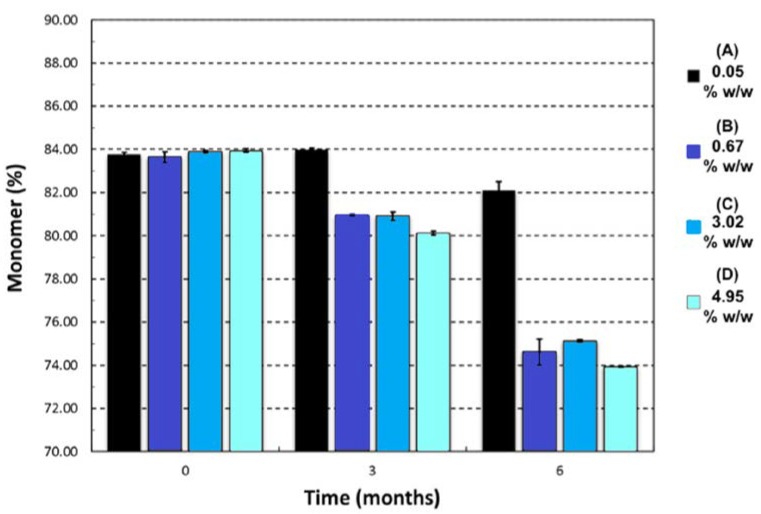
Change in monomer (%) after 6 months of storage at 45 °C for different initial moisture samples of IgG 50 mg/mL (A = 0.05% ± 0.01% *w*/*w*, B = 0.67% ± 0.14% *w*/*w*, C = 3.02% ± 0.06% *w*/*w* and D = 4.95% ± 0.40% *w/w*). Monomer content was stable for −20 and 20 °C at 84.0% ± 0.5% Error bars represent 95% confidence intervals (*n* = 3 vial replicates).

**Figure 7:**
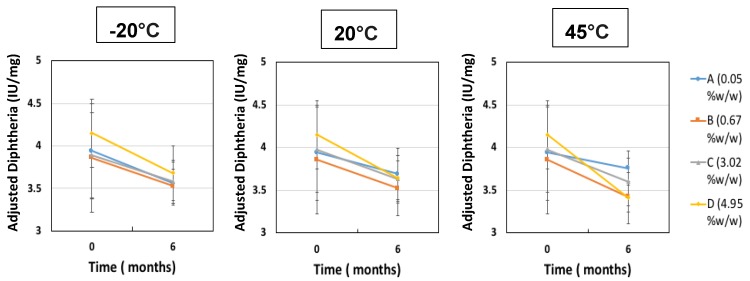
ELISA Diphtheria binding activity (IU/mL) after 6 months of storage for different initial moisture samples of FD IgG 50 mg/mL (A = 0.05% ± 0.01% *w*/*w*, B = 0.67% ± 0.14% *w*/*w*, C = 3.02% ± 0.06% *w*/*w* and D = 4.95% ± 0.40% *w*/*w*) at −20 °C, 20 °C and 45 °C. Error bars represent 95% confidence intervals.

The second investigation considered the acceptable “moderate” moisture content of FD IgG at 50 mg/mL over the course of a 6 months stability study. Samples were all prepared initially in a low moisture cycle producing moisture contents of 0.05% ± 0.01% *w*/*w* (*n* = 3), (A). Vials were exposed to the humid room atmosphere at several different time intervals of 30, 90 and 180 min, respectively (57% RH at temperature of 20 °C). The increased moisture contents before storage were measured as (B) 0.67% ± 0.14% *w*/*w*, (C) 3.02% ± 0.06% w/w and (D) 4.95% ± 0.40% *w*/*w* (*n* = 3). Figure 5 shows the moisture ingress over 6 months from different initial starting moisture contents. All samples showed constant moisture content at −20 °C; however, the drier the sample had started out then the more moisture ingress was observed after 6 months at elevated storage temperatures. Sample A (with the lowest initial moisture content) had a significant increase in moisture content after 6 months at 20 °C and 45 °C up to 1% *w*/*w*. For Sample A, this uptake is most likely to reflect moisture transfer from inside the vial stopper. For very low moisture content FD materials, there is a diffusion-driven water migration from the stopper interior to the relatively dry cakes. The other samples have moisture contents within an experimental error of each other and exhibited no similar major moisture ingress. The effect of different moisture content on the monomer content (%) and binding activity (IU/mL) is shown in Figure 6 and Figure 7. Table 3 shows the comparison between moisture content of samples and the resulting measured T_g_. Over the course of 6 months, storage at −20 °C to 20 °C increased the moisture content up to 5% *w*/*w* and had no impact on monomer content or binding activity. However, at 45 °C, just after 6 months storage, the samples with higher moisture (above 1% *w*/*w* moisture) began to see around a 10% drop in monomer content, whereas for sample A (below 1% *w*/*w* moisture) there is no losses. This is in stark contrast with the binding activity results, which show that at no point is there any loss for all moisture content samples (A–D) up to 5% *w*/*w*. One possible explanation for this behaviour is that in the previous case study moisture content ingress naturally increased over time, whilst for this study the samples were exposed to atmosphere and hence possible oxygen trapped in headspace. Oxidation might have occurred in this study, which could have led to more protein–protein aggregation.

These monomer and binding activity results from the induced exposure moisture seemed to suggest that modest moisture content (≈3% *w*/*w*) was perfectly acceptable for 50 mg/mL IgG when stored at up to 20 °C. However, from the results in this study there was no Gaussian-shaped distribution in terms of moisture and stability observed. In fact, a more linear correlation with moisture was observed for higher temperature storage, which was more in line with what Pikal et al. had observed in a moisture-based stability study [14]. It is worth noting that Chang et al. in a study on a Mab also reported that there was better stability at 2–3% moisture content than for drier samples [29]. This supports our finding here that an optimum moisture content exists and should be investigated for each formulation.

### 3.3. Case Study 3: Stability of Induced “Low” Versus “High” Moisture FD Cycles

**Figure 8:**
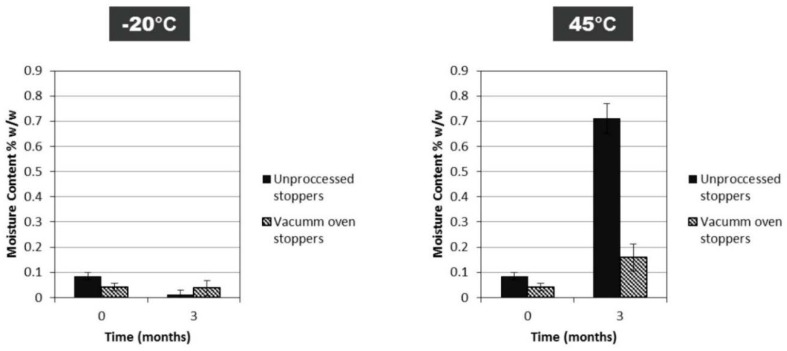
Difference in moisture content for FD IgG (50 mg/mL) between vials with vacuum-oven-dried stoppers and vials with untreated stoppers straight out of packaging after 3 months of storage at −20 °C and 45 °C (*n* = 3 vial replicates).

In Case Study 3, we looked at the effect of FD cycle-induced moisture content between a very low moisture content and a moderately high one for a single IgG concentration (50 mg/mL). Residual moisture content is controlled by the shelf temperature used in secondary drying. Post-drying exposure to controlled humidity was used in Case Study 2. Cycle-induced moisture might provide different data results with regard to moisture content. The low moisture cycle produced cakes with 0.11% ± 0.01% *w*/*w*, while the high moisture cycle produced cakes with 3.02% ± 0.8% *w*/*w* (*n* = 3). Previously it has been described that a much higher moisture contents(8% w/w) decreased the stability of a MAbs dried at a 40 mg/mL concentration [11]. However, here, more representative moisture contents (0.1% *w*/*w* for low cycle and 3% *w*/*w* for high cycle) within the acceptable moisture range for intravenous immunoglobulin [8] were engineered by control of the secondary drying temperature conditions. The moisture content changes from the initial starting points over 12 months of storage. The samples from the low moisture cycle batch had an increase to about 1.5% *w*/*w* moisture at 20 °C and 45 °C storage. The samples from the high moisture batch, however, exhibited no such moisture ingress across the temperature range. This may illustrate an equilibration of the moisture in the closure and the hygroscopic dry FD product. Matejtschuk et al. [30] had observed similar equilibrating moisture effects in vials in a study with a model HSA formulation. Once again, a moisture increase over time is seen for vials at or above 20 °C, especially for FD samples that initially had a very low moisture content. It was most likely that a significant portion of moisture ingress could be arising from the stoppers themselves (Figure 8). Stopper drying has been effectively used in the past to maintain a low moisture content during storage [31], and can help reduce water ingress and potential degradation at higher storage temperatures.

**Figure 9:**
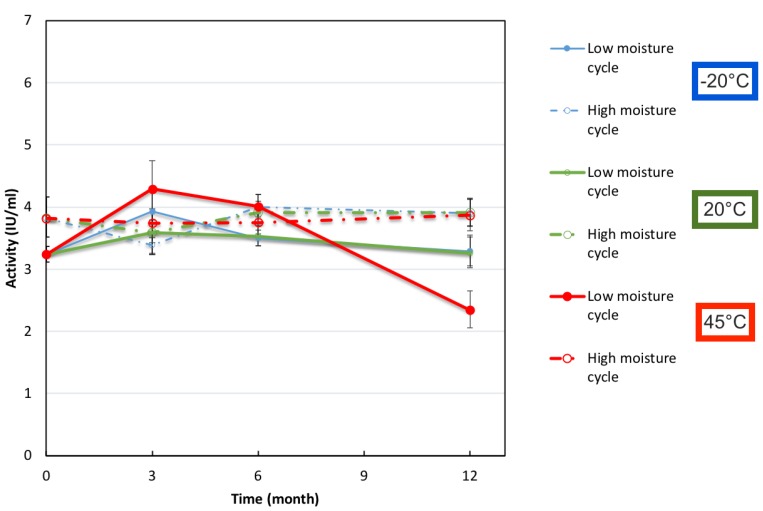
Diphtheria Binding Activity determined by ELISA for FD IgG 50 mg/mL in “low” or “high” moisture cycle batches over 12 months of storage. Error bars represent 95% confidence intervals.

**Figure 10:**
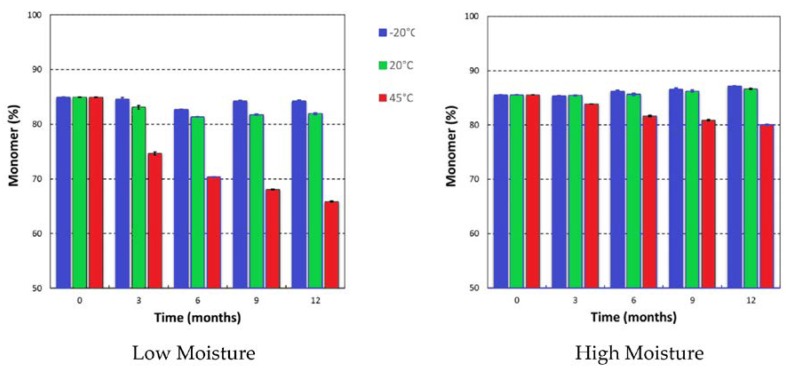
Change in monomer content (%) of FD IgG 50 mg/mL vials made under “low” or “high” moisture cycles over 12 months of storage. Error bars represent 95% confidence intervals (*n* = 3).

Figure 9 and Figure 10 shows the binding activity and monomer (%) content and compared for IgG (50 mg/mL) at both a high and low moisture content. After 12 months, there is no major difference or drop in monomer content at −20 °C and 20 °C for vials in either a low cycle or high cycle batch moisture content (remains steady above 80% monomer content). Similarly, the binding activity does not change greatly for either low and high cycle batch vials at −20 °C and 20 °C storage. However, at 45 °C, samples from the low moisture cycle were observed to have a significantly greater drop in monomer content compared to samples from the high moisture cycle batch. This was also seen for the binding activity data, where the low moisture sample vials saw a drop with activity at 45 °C, whereas the high moisture vials did not. This is also in contrast to the previous Case Study 2, where the samples with highest moisture saw greatest monomer drop at elevated temperature of 45 °C. As stated previously, one possible explanation for this is that the vials in this study were stoppered under vacuum and nitrogen gas, while in the other study the vials were exposed to the environment and had oxygen trapped in the headspace. Oxidation might have occurred in the previous case study, which would have induced pathways to lead to more protein–protein aggregation. However, in a previous study we did not observe any detriment in antibody activity on storage from a higher oxygen content in the headspace [32]. This might also be further evidence of over-drying and for water replacement theory with some moisture being essential for the FD cake stability in allowing hydrogen bonds to form.

Previous reported research from Wang [33], Liu et al. [34] and Greiff [13] have all described an increase in aggregation with higher moisture content (over the optimum level). However, some past studies have also shown that acceptable moisture contents may be permissible in FD cakes without effecting stability [5]. The results here showed evidence that over drying can also have a detrimental effect on stability. In another alternate study, Wang showed for a different formulation that over-drying had greater loss in activity and increased aggregation. Over-drying of FD insulin has also showed to cause damage and increased product degradation [35]. Hubbard et al. noted how a FD reference plasma, with an optimised formulation and a modest moisture content, had greater stability than one with a very low moisture content [36]. These results show some confirmation of the water replacement theory. If the FD IgG samples are over dried, the hydrogen bonds are lost between the protein and the water, hence you have a change in conformational structure. Some moisture may be crucial to maintaining the stability of the FD biologics.

**Figure 11:**
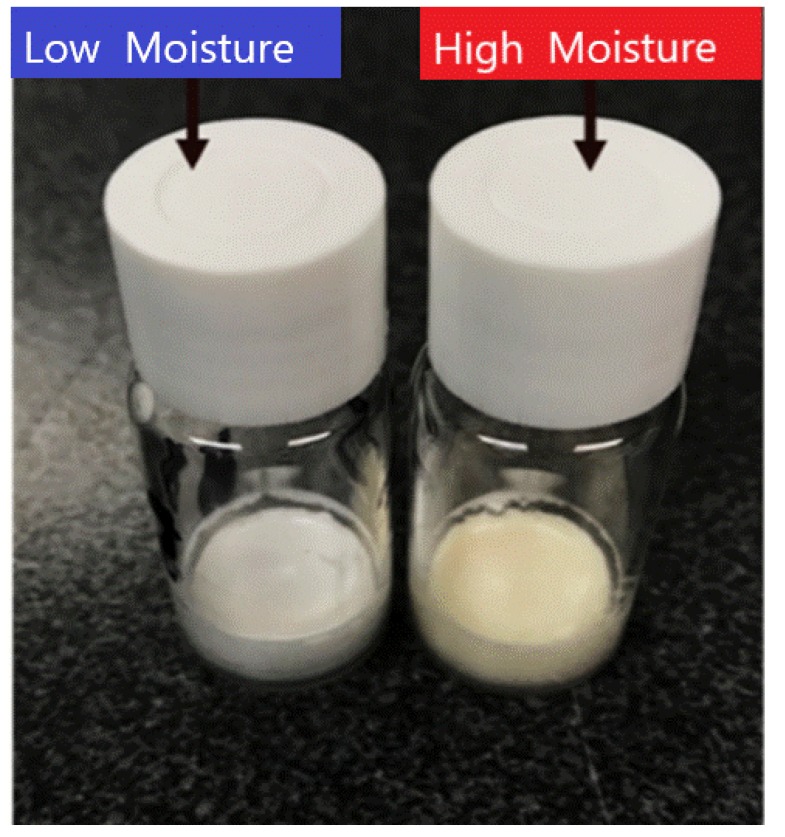
Comparison of the visual appearance between vials FD in either “low” or “high” moisture cycles for FD IgG (50 mg/mL) after 12 months storage at 45 °C.

These results suggest that in this model system the higher moisture (3% *w*/*w*) samples are as good as the extremely low moisture samples (<1% *w*/*w*) and might even show reduced monomer/activity loss at elevated temperatures. Upon visual inspection, there was no colour change between the high and low moisture vials at −20 °C and 20 °C for the entire storage duration. However, at 45 °C a visible yellow discolouration had appeared on the cakes with the higher moisture content (Figure 11). The discolouration present in the higher moisture batch vials was probably due to Maillard browning (caused by protein free amino group reaction with sugars). Schüle et al. also saw a brown discoloration with lactose in their dried IgG1 stabilization study, which they attributed to Maillard reactions [37]. Kanojia et al. found that after accelerated thermal treatment a chemical modification had occurred in their dry sucrose-dextran IgG formulations, which they too postulated was due to Maillard reaction products [38]. One explanation then might be that there was reducing sugar impurities present in the formulation; however, this is very unlikely due to using a pharmaceutical-grade material. This study also used sucrose in the formulation, which is not a reducing sugar. However, while sucrose is not a reducing sugar, it can break down to fructose and glucose, which are reducing sugars; so, this may explain why this discoloration was only observed for samples stored at higher temperature [39]. Matejtschuk et al. spotted similar discolouration for formulations containing sucrose at elevated storage temperatures [40]. The presence of high moisture content further facilitates these reactions and results in a change in the appearance. It is also a possibility that at the formulated sucrose concentration, buffer crystallisation could have occurred during the freezing, thereby leading to a change to low pH at which sucrose could have broken down to fructose and glucose [41]. Of course, when it comes to commercialisation, product appearance is a key factor and acceptable and unacceptable product appearance defects have recently been reviewed [42]. Although it may be possible to illustrate that a relatively higher moisture content is beneficial in terms of product stability, the risk of developing a yellow cake appearance on storage would be a drawback for customers as most would be familiar with a white homogeneous product appearance. The potential immunogenicity effects would also need to be addressed.

## 4. Conclusions

Freeze-drying can provide excellent long-term stability for high concentration IgG proteins but needs to be considered in the context of factors such as moisture content and intended storage temperature. FD IgG from 10–200 mg/mL had minimal monomer/activity losses at up to 12 months of storage at 20 °C. IgG 10 mg/mL with the 1:1 protein:sucrose ratio had greater stability shielding at elevated temperatures compared to concentrations above 50 mg/mL, but this may reflect the need for a higher sucrose content at a higher protein composition. There may be an optimum moisture content range for FD IgG, and this needs to be informed with reference to the storage temperature compared to the glass transition temperature. From −20 °C to 20 °C moisture content up to 5% *w*/*w* does not seem to have any impact on monomer or binding activity during prolonged storage. It is only at elevated temperatures, such as at 45 °C storage, that high moisture seemed to affect the monomer for IgG at 50 mg/mL, although this might be because of oxygen ingress during the exposure trial study. In the comparison between “high” versus “low” FD cycle-prepared materials the opposite was seen, in that the higher moisture content vials performed better at 45 °C than the extremely dry moisture samples. While some moisture content might be permissible and in fact even beneficial, Maillard reactions can occur (discolouration), which could affect commercial prospects for high moisture content samples aged at 45 °C. Moisture ingress into the FD cakes was observed to occur in every trial reported here, and is especially the case for drier samples and at more elevated storage temperatures, although one potential way of counteracting this effect could be by vacuum heat treating the rubber stoppers [31].

## Figures and Tables

**Table 1:** Freeze drying cycle for IgG formulations.

Cycle	Freezing Temperature (°C)	Freezing Ramp Rate (°C/min)	Freezing Hold Time (min)	Primary Drying Temperature (°C)	Primary Hold Time (min)	Secondary Drying Temperature (°C)	Secondary Drying Ramp Rate (°C/min)	Secondary Hold Time (min)
Low Moisture	−40	1.00	120	−15	1200	30	0.15	600
High Moisture	−40	1.00	120	−40	900	15	0.18	60

**Table 2:** Comparison of difference in storage temperature **T** and glass transition temperature **T_g_** after 12 months of storage.

Storage Temperature (°C)	IgG Concentration (mg/mL)	T_g_	T_g_-T (°C)
−20	10	101	121
−20	50	104	124
−20	100	110	130
−20	200	105	125
20	10	94	74
20	50	96	76
20	100	117	97
20	200	116	96
45	10	77	32
45	50	84	39
45	100	84	39
45	200	90	45

**Table 3:** Comparison of difference in storage temperatures and glass transition temperatures after 6 months of storage.

Temperature (°C)	Initial Moisture Sample (% *w*/*w*)	T_g_	T-T_g_ (°C)
−20	0.05 (A)	115	135
	0.67 (B)	113	133
	3.02 (C)	113	133
	4.95 (D)	111	131
20	0.05 (A)	114	94
	0.67 (B)	111	91
	3.02 (C)	111	91
	4.95 (D)	110	90
45	0.05 (A)	112	67
	0.67 (B)	113	68
	3.02 (C)	110	65
	4.95 (D)	108	63
